# Therapeutic Potential of Gene‐Modified MSC‐Exos in Insulin Resistance: Mitochondrial Crosstalk and miRNA Regulation

**DOI:** 10.1111/jcmm.71342

**Published:** 2026-09-04

**Authors:** Qinghua Jia, Bing Li, Yuan Xing, Yuanyuan Zhang, Baodong Gao, Xiangying Li, Xiaoqin Ha

**Affiliations:** ^1^ Department of Clinical Laboratory The 940th Hospital of Joint Logistics Support Force of Chinese People's Liberation Army Lanzhou Gansu China; ^2^ Key Laboratory of Stem Cells and Gene Drugs of Gansu Province Lanzhou Gansu China

**Keywords:** gene modification, insulin resistance, mesenchymal stem cells, exosomes, microRNA, mitochondrion, precision medicine, type 2 diabetes mellitus

## Abstract

Insulin resistance (IR) is the key driver of type 2 diabetes mellitus, metabolic dysfunction‐associated steatotic liver disease, and cardiometabolic disorders. Mesenchymal stem cell‐derived exosomes (MSC‐Exos) as a potent cell‐free alternative can deliver miRNAs, proteins, and mitochondrial regulators to reactivate the IRS1/PI3K/Akt/GLUT4 axis. Beyond restoring insulin signalling, these vesicles can protect β‐cells, alleviate endoplasmic reticulum stress, rescue mitochondrial mitophagy and respiration. The genetic engineering strategies including improving the expression of miR‐21, miR‐3075, or Sirtuin‐3, and silencing miR‐29b‐3p amplify the therapeutic efficacy. However, scalable good manufacturing practice production, long‐term safety of modified products, and advanced therapy medicinal product regulation are still needed to overcome. This review will introduce the potential of gene‐modified MSC‐Exos and miRNAs targeting insulin signalling and mitochondrial rescue, which have shown a shift from symptomatic relief to precision disease‐modifying therapy for IR.

## 1 Introduction

Insulin resistance (IR) acts as a pathophysiological foundation of major metabolic disorders, such as type 2 diabetes mellitus (T2DM), non‐alcoholic fatty liver disease, obesity and metabolic syndrome [1]. The responsivity of insulin‐sensitive tissues to physiological levels of circulating insulin is impaired, such as skeletal muscle, adipose tissue and liver, which results in reducing glucose uptake, dysregulated lipid metabolism and compensatory hyperinsulinemia [2]. IR causes detrimental systemic effects and fosters a state of chronic inflammation and vascular endothelial impairment, leading to heightened cardiovascular risk and presenting a substantial burden on global public health.

The defects in insulin signalling cascade drive IR. The autophosphorylation and subsequent tyrosine phosphorylation of insulin receptor substrates (IRS) triggered by insulin binding to its receptor consequently activate the phosphoinositide 3‐kinase (PI3K)/Akt pathway and drive translocation of glucose transporter 4 (GLUT4) to the plasma membrane in muscle and adipose tissue [3]. In IR states, this pathway is interfered with by damaged tyrosine phosphorylation and excessive serine phosphorylation of IRS and downstream inhibition of PI3K/Akt signalling, finally diminishing glucose uptake and utilization [4]. Mitochondrial dysfunction, excessive reactive oxygen species (ROS) production, persistent inflammation, and ectopic lipid accumulation in non‐adipose tissues further aggravate insulin signalling defects [5]. Initially, pancreatic β‐cells offset through increasing insulin secretion; however, chronic stress induces β‐cell exhaustion and apoptosis, perpetuating hyperglycemia and disease progression [6].

The aim of conventional pharmacotherapies like metformin and thiazolidinediones for IR is primarily to reverse systemic glucose homeostasis and modestly improve insulin sensitivity [7]. However, these agents frequently lack the capacity to adequately handle mitochondrial impairment, inflammatory signalling, and epigenetic alterations [8]. Recent research has turned attention to the novel therapeutic strategies of targeting the multifaceted cellular and molecular drivers of IR.

Mesenchymal stem cells (MSCs) have been regarded as prospective therapy candidates because of their potent paracrine and immunomodulatory properties. The therapeutic advantages of MSCs are largely mediated by secreted factors rather than depending on direct differentiation or engraftment. Mesenchymal stem cell‐derived exosomes (MSC‐Exos) are 30–150 nm nanovesicles, which play a crucial role [9]. They encompass microRNAs (miRNAs), proteins, and lipids; meanwhile, they modulate gene expression, metabolic pathways, and cellular function in recipient tissues [9]. Additionally, MSC‐Exos can improve insulin sensitivity, attenuate systemic inflammation, and recover metabolic homeostasis across diverse models of IR [10].

The mechanism of mitochondrial crosstalk has received significant attention. MSC‐Exos can act as vectors for mitochondrial transfer, bioenergetic balance recovery, and oxidative burden reduction in target cells, which activate cellular bioenergetics, limit oxidative damage, and enhance metabolic efficiency [11]. These findings provide support for combinatorial therapies. Genetical modified MSCs‐Exos with specific miRNAs or mitochondrial regulators supply an approach to simultaneously rectify insulin signalling defects and organelle‐level impairments in IR tissues.

Overall, gene‐modified MSC‐Exos mark a paradigm shift in IR treatment by simultaneously targeting molecular, cellular, and organelle‐level abnormalities. Here, we outline the pathogenesis of IR with mitochondrial impairment, followed by an evaluation of MSC‐Exos' therapeutic potential. We probe into the dual mechanisms of miRNA regulation and mitochondrial rescue, and characterize genetic engineering strategies designed to optimize exosome payloads. The review summarizes a critical interpretation of the clinical translational hurdles and prospective research avenues.

## 2 Pathophysiology of IR: Focus on Mitochondria and Inter‐Organ Crosstalk

### 2.1 Major Mechanisms of IR

The main pathogenesis of IR is that the peripheral tissues are incapable of efficiently disposing glucose. In physiological state, insulin starts to involve receptor autophosphorylation and tyrosine phosphorylation of IRS so that it transports GLUT4 to the cell surface by activating PI3K/Akt axis [12]. In the IR state, however, this cascade is undermined by a series of molecular defects. The inhibition of IRS tyrosine phosphorylation and stress kinase‐mediated serine phosphorylation (e.g., JNK, PKC) can collectively suppress PI3K/Akt activity [13]. Additionally, the signalling network is further repressed by negative regulators like PTEN and SOCS proteins, and a chronic inflammatory milieu full of cytokines such as TNF‐α and IL‐6 [14].

Simultaneously, the buildup of diacylglycerols and ceramides in non‐adipose tissues creates a lipotoxic environment. This triggers a cascade of endoplasmic reticulum stress and oxidative damage, which collectively amplifies sustained low‐grade inflammation [15]. In adipose tissue, adipocytes exhibit macrophage infiltration with M1 polarization, reduced adiponectin secretion, and increased release of leptin and resistin, finally amplifying systemic IR [16].

### 2.2 Mitochondrial Dysfunction in IR

Mitochondria are pivotal regulators of cellular energy metabolism, substrate oxidation, and ROS homeostasis. In the context of IR skeletal muscle, the decline in mitochondrial oxidative capacity consistently declines [17]. This deficiency obstructs fatty acid oxidation and the buildup of partially oxidized lipid intermediates; meanwhile, these metabolites sabotage insulin signalling through the activation of PKC isoforms and JNK [17]. In the liver, promoting pathological gluconeogenesis and lipid accumulation are further exacerbated by mitochondrial dysfunction [18].

In addition to impaired respiratory capacity, IR is accompanied by mitochondrial derangements. These include disrupted fusion/fission dynamics, stalled mitophagy, and blunted biogenesis due to downregulation of the PGC‐1α/NRF1/TFAM axis [19]. Such defects induce a self‐reinforcing loop of ROS overproduction and ATP depletion. This pathological feedback intensifies insulin signalling defects; furthermore, it provokes β‐cell failure under prolonged metabolic strain [20].

### 2.3 Inter‐Organ Mitochondrial Crosstalk and Exosome/EV‐Mediated Communication

The intercellular and inter‐organ communication is important to the systemic feedback of IR and its therapeutic modulation. Exosomes from adipose tissue can deliver miRNAs and other cargo to skeletal muscle and liver, thereby modulating local insulin sensitivity and inflammatory tone [21]. More remarkably, MSCs and certain other cell types have been shown to transfer functional mitochondria to recipient cells via tunnelling nanotubes (TNTs), gap junctions, and extracellular vesicles (EV) [22].

Although most direct evidence of mitochondrial transfer originates from models of acute tissue injury (e.g., ischemia–reperfusion, lung injury), the concept is highly related to chronic metabolic diseases [23]. In IR, mitochondrial impairment influences muscle, liver, adipose tissue, and pancreatic islets, thereby, strategies that restore mitochondrial function through intercellular mitochondrial delivery or exosome‐mediated transfer of mitochondrial regulators offer an attractive therapeutic approach [24]. The crosstalk mechanisms can remotely rescue of bioenergetic capacity and mitigation of oxidative stress in metabolically stressed tissues.

## 3 MSC‐Derived Exosomes and IR

### 3.1 Features of MSC‐Exosomes

MSCs are multipotent stromal cells harvested from various tissues, such as bone marrow, adipose tissue, umbilical cord, placenta, and dental pulp [25]. Their therapeutic potential is largely driven by the paracrine secretion of EV, especially exosomes, which are the primary active agents within the MSC secretome [26].

MSC‐Exos are small membrane‐bound vesicles generated through the endosomal pathway [27]. The luminal cargo of MSC‐Exos comprises a heterogeneous mixture of bioactive molecules, including miRNAs, long non‐coding RNAs, proteins, lipids, and metabolites [27]. They carry specific surface markers, notably tetraspanins (CD9, CD63, CD81) and endosomal sorting complex required for transport‐associated proteins (TSG101, ALIX) [28]. Their lipid bilayer is rich in sphingomyelin and cholesterol, so it is critical for maintaining vesicle stability and supporting biogenesis [29]. When recipient cells are taken up through endocytosis, phagocytosis, or direct membrane fusion, these cargos regulate target gene expression, signalling pathways, and cellular phenotypes [30].

Compared with whole‐cell MSC transplantation, exosome‐based approaches offer several key advantages. There is negligible risk of tumorigenicity or ectopic differentiation, low immunogenicity, ease of storage and standardization as an off‐the‐shelf biologic product, and the ability to cross biological barriers including the blood–brain barrier [31]. These attributes position MSC‐Exos as a highly attractive cell‐free platform for the treatment of IR.

### 3.2 MSC‐Exos in IR Preclinical Evidence

A growing body of preclinical studies demonstrate that MSC‐Exos can improve insulin sensitivity and ameliorate metabolic dysfunction in multiple insulin target tissues, including skeletal muscle, adipose tissue, liver, and pancreatic islets [32, 33] (Table 1).

**TABLE 1:** Representative studies of MSC‐derived exosomes in IR.

Source MSC and exosome type	Model (in vitro/in vivo)	Major effects/mechanisms	References
hUC‐MSC‐Exos	Rat T2DM (HFD + STZ)	↑ GLUT4 translocation, ↓HbA1c, ↑ glucose uptake	[34]
hUC‐MSC‐Exos	Human insulin‐resistant adipocytes	↑ SIRT1 & IRS1, ↓ leptin, ↑ adiponectin, ↑ glucose uptake	[35]
BMSC‐Exos	HFD‐induced obese mice & 3T3‐L1 adipocytes	↑ GLUT4, activation of PI3K/AKT, ↓ M1 macrophages, ↓ TNF‐α/IL‐6	[36]
hWJMSC‐Exos	HFD mice with hepatic steatosis	↑ FGF21/adiponectin axis, ↓ endoplasmic reticulum(ER) stress & lipotoxicity, ↑ lipid oxidation	[37]
hUC‐MSC‐Exos	Rat T2DM (HFD + STZ)	↓ β‐cell apoptosis, ↑ GLUT4, ↑ insulin secretion, ↑insulin/AKT signalling pathway	[38]

Exosomes derived from human umbilical cord MSCs (hUC‐MSC‐Exos) administered to high‐fat diet (HFD) plus streptozotocin‐induced diabetic mice significantly enhanced insulin sensitivity, as evidenced by increased insulin receptor substrate1 (IRS1) and Akt phosphorylation, enhanced GLUT4 membrane translocation, and improved glycogen storage in liver and skeletal muscle [39]. In vitro, hUC‐MSC‐Exos restored glucose uptake in insulin‐resistant human adipocytes by upregulating SIRT1 and IRS1 expression and normalizing adipokine profiles (decreased leptin, increased adiponectin) [38].

Bone marrow MSC‐derived exosomes (BMMSC‐Exos) have been shown to attenuate IR in diet‐induced obese mice and in 3T3‐L1 adipocytes by activating the PI3K/Akt pathway, increasing GLUT4 expression, and shifting adipose tissue macrophage polarization toward an anti‐inflammatory M2 phenotype [36]. Similarly, Wharton's jelly MSC‐derived exosomes (WJ‐MSC‐Exos) alleviated hepatic steatosis and IR in obese models by activating the FGF21/adiponectin axis, reducing ER stress and lipotoxicity, and enhancing mitochondrial fatty acid oxidation [40].

Collectively, these studies indicate that MSC‐Exos exert therapeutic effects through both direct enhancement of insulin signalling in target cells and indirect modulation of inter‐organ metabolic and inflammatory crosstalk.

However, most of these preclinical findings are derived from short‐term HFD or STZ‐induced rodent models with relatively small sample sizes. Significant heterogeneity exists across studies in MSC sources, exosome isolation methods, and dosing regimens, which limits the generalizability and clinical translatability of the observed effects.

### 3.3 Mechanisms of Action in IR

#### 3.3.1 Enhancement of Insulin Signalling

MSC‐Exos not only enhance the insulin signalling cascade by amplifying the activity of IRS1, PI3K, and Akt but also enhance tyrosine and serine or threonine phosphorylation [41]. This molecular intervention triggers GLUT4 translocation to the plasma membrane in skeletal muscle; thereby, it enhances glucose uptake and glycogen synthesis [42]. In both HFD mouse models and IR cell models, these vesicles reverse signalling impairments, normalize GLUT4 expression, and stabilize systemic glucose homeostasis [43].

However, current evidence mainly focuses on in vitro small animal models. There is a notable absence of long‐term data from large animals or translational human systems, so that the true therapeutic impact of MSC‐Exos on human insulin signalling is unproven.

#### 3.3.2 Anti‐Inflammatory Effects

Exosomal miRNAs like miR‐146a and miR‐21 act as critical inhibitors of NF‐κB signalling and its downstream inflammatory cascades [44, 45]. By suppressing the release of pro‐inflammatory cytokines such as TNF‐α, IL‐6, and IL‐1β, these miRNAs drive a phenotypic switch from a pro‐inflammatory M1 state to an anti‐inflammatory M2 profile in adipose tissue macrophages [46]. This repolarization alleviates the chronic, low‐grade inflammation that underpins obesity‐induced IR [47]. Furthermore, MSC‐Exos have been shown to target the TLR4/NF‐κB axis, extending their anti‐inflammatory influence to other insulin‐resistant tissues [48].

However, these anti‐inflammatory findings are largely derived from acute or subacute inflammation models. Critical questions regarding the durability, dose–response dynamics, and clinical relevance of exosomal miRNA‐mediated macrophage polarization in chronic obesity‐related inflammation remain unanswered.

#### 3.3.3 Regulation of Adipokine Secretion

MSC‐Exos re‐establish adipokine equilibrium by boosting adiponectin secretion while simultaneously suppressing leptin, thereby functioning as endocrine modulators that improve systemic insulin sensitivity [21, 38]. This metabolic benefit is driven by the activation of the FGF21‐adiponectin axis and AMPK‐SIRT1‐PGC‐1α signalling, which effectively mitigates adipocyte hypertrophy and prevents ectopic lipid deposition in the obesity model [49]. Consequently, the normalization of adipokine profiles dampens inflammation and fosters improved metabolic regulation across peripheral tissues.

#### 3.3.4 Alleviation of ER Stress and Lipotoxicity

MSC‐Exos alleviate ER stress of hepatocyte marked by reduced CHOP and GRP78 levels as well as promote lipid catabolism by boosting PPARα and CPT1A expression [50]. This intervention is mediated by autophagy improvement or AMPK pathway activation in order to restore hepatic insulin sensitivity [51]. Consequently, these changes ameliorate steatosis and optimize glucose metabolism in diet‐induced obesity and metabolic dysfunction‐associated steatotic liver disease.

#### 3.3.5 Protection of Pancreatic β‐Cells

miR‐21 and miR‐146a in exosome act as critical safeguards against oxidative and ER stress‐induced β‐cell apoptosis, primarily by modulating FasL, PDCD4, and p38 MAPK phosphorylation [52]. This protective mechanism mainly is related to maintenance of β‐cell viability and insulin secretory capacity, and prevention of dedifferentiation [53]. In hypoxic or diabetic environments, miR‐21 specifically downregulates ER stress markers like GRP78 and CHOP to inhibit apoptosis, and miR‐146a ameliorates cellular function through targeting NUMB/β‐catenin axis [54, 55].

#### 3.3.6 Improvement of Mitochondrial Function

Through the delivery of miRNAs and proteins like miR‐30 and miR‐494, MSC‐Exos correct mitochondrial deficits in metabolic tissues plagued by insulin resistance [56]. By stimulating biogenesis and restoring oxidative phosphorylation, these vesicles curtail excessive ROS production and boost ATP synthesis. This metabolic rescue is driven by the activation of PGC‐1α and SIRT1 pathways, effectively counteracting the mitochondrial impairment caused by lipotoxicity and inflammation [57].

However, current evidence regarding mitochondrial improvement by MSC‐Exos is largely indirect (mainly through miRNA or protein regulation). Direct mitochondrial transfer and long‐term bioenergetic rescue in insulin‐resistant tissues are still weakly supported, and tissue‐specific differences in efficacy have not been sufficiently clarified.

## 4 miRNA Regulation by MSC‐Exos in IR

### 4.1 miRNAs and IR

miRNAs are small, endogenous, non‐coding RNAs (~22 nucleotides) that post‐transcriptionally regulate gene expression through miRNA degradation or translational repression [58]. They serve as critical fine‐tuners of metabolic homeostasis, and dysregulation of specific miRNAs has been strongly implicated in the pathogenesis of IR and T2DM [59].

In IR states, several miRNAs target key nodes of the insulin signalling pathway (e.g., IRS1/PI3K/Akt axis), GLUT4 expression, lipid metabolism, mitochondrial biogenesis, and inflammatory cascades [60]. Well‐characterized examples include miR‐29 family members, miR‐34a, miR‐143, miR‐320, and miR‐144, which collectively promote signalling defects, ectopic lipid accumulation, and chronic inflammation [61]. Given their stability and ability to be encapsulated in EV, miRNAs within exosomes represent potent mediators of intercellular communication, enabling remote modulation of insulin sensitivity across metabolic organs [62].

### 4.2 Key miRNAs in MSC‐Exos Relevant to IR

MSC‐Exos naturally carry a diverse miRNA cargo capable of influencing insulin signalling, mitochondrial function, and inflammatory tone in target cells. Several miRNAs recurrently associated with MSC‐Exos have been linked either directly or mechanistically to amelioration or exacerbation of IR (Table 2).

**TABLE 2:** miRNAs in exosomes from different sources and functional roles in IR.

miRNA	Source and biological context	Targets/Signalling pathways	Functional effects in IR	References
miR‐29b‐3p	BMSC‐Exos	SIRT1	Induces IR; impaired or enhanced insulin sensitivity	[63]
miR‐21	hUC‐MSC‐Exos, β‐cell stress models	p38, MAPK	Anti‐apoptotic, protection of β‐cells	[55]
miR‐3075	hepatocyte‐derived exosomes	Fatty acid 2‐hydroxylase	Enhances insulin sensitivity	[64]
miR‐210	Adipose tissue macrophages derived exosomes	SIDT2	Enhances IR	[65]

#### 4.2.1 miR‐29b‐3p

Exosomes from aged bone marrow mesenchymal stem cell (BMMSC) are enriched in miR‐29b‐3p, which directly targets SIRT1, a key regulator of mitochondrial function and insulin signalling, thereby promoting metabolic dysfunction in adipocytes, hepatocytes, and skeletal myocytes [63]. Inhibition of miR‐29b‐3p in these exosomes abolishes the detrimental effects, rescuing insulin sensitivity and glucose uptake. This highlights miR‐29b‐3p as a senescence‐associated miRNA that may contribute to age‐related IR [63].

#### 4.2.2 miR‐21

Exosomes from human umbilical cord MSCs contain miR‐21, which exerts protective effects on pancreatic β‐cells under hypoxic, ER stress, and pro‐inflammatory conditions [32]. miR‐21 suppresses pro‐apoptotic pathways like PTEN/PI3K/AKT, p38, and MAPK, thereby preserving β‐cell viability and function [55, 66]. Given the central role of β‐cell loss in the progression from IR to T2DM, miR‐21‐enriched MSC‐Exos represent a promising strategy for β‐cell preservation.

#### 4.2.3 miR‐3075

miR‐3075 as an insulin‐sensitizing miRNA enriched in hepatocyte‐derived exosomes during early‐stage obesity (4‐week HFD), where it enhances insulin sensitivity in adipocytes, myocytes, and hepatocytes by targeting fatty acid 2‐Hydroxylase (FA2H) [64]. Depletion of FA2H mimics the beneficial effects of miR‐3075. In contrast, this compensatory mechanism is lost in chronic obesity, where hepatocyte exosomes promote IR [64]. Although direct evidence of high miR‐3075 enrichment in MSC‐Exos remains limited, targeted overexpression of miR‐3075 in MSCs could represent a rational engineering strategy to confer insulin‐sensitizing properties to exosomes [64].

#### 4.2.4 miR‐210

miR‐210, a hypoxia‐responsive mitochondrial miRNA, modulates mitochondrial respiration and metabolic flexibility [65]. Exosomal transfer with miR‐210 has been implicated in regulating genes involved in mitochondrial complex assembly and oxidative metabolism [67]. Its effects appear context‐dependent: moderate levels may support adaptive metabolic responses, whereas sustained overexpression could impair glucose utilization. Future research should define the function and therapeutic efficacy of MSC‐Exos.

### 4.3 Implications for Gene‐Modified MSC‐Exos

The metabolic activity of miRNAs from MSC‐Exos pinpoints their value as delivery vehicles for IR therapy [68]. However, current main research confines to mechanistic exploration. Since certain candidates like miR‐3075 exhibit low natural enrichment and context‐dependent activity, therapies depending on a single miRNA species may affect the consistent results in different disease stages. Fortunately, gene‐modified MSC‐Exos permit precise cargo modification, so it creates opportunities to amplify therapeutic benefits while counteracting detrimental signals [68].

Genetic engineering uses lentiviral transfection or electroporation methods in order to ensure the precise modification of exosomes [69]. By enriching exosomes with therapeutic molecules while silencing harmful factors, which generates significantly more specific and superior clinical outcomes [70].

MiR‐21 protects β‐cells by inhibiting PDCD4, FasL, p38, and MAPK [55]. MiR‐146a attenuates inflammation by blocking NF‐κB and reducing the secretion of TNF‐α, IL‐6, and IL‐1β [71]. Additionally, engineered miR‐3075 mimics can improve insulin sensitivity across metabolic tissues in an early onset obesity model [64]. The enrichment of exosomes with these miRNAs has a synergistic effect, which propagates their regulatory impact on insulin signalling, inflammation, and β‐cell function [72].

Notably, Sirtuin‐3 (SIRT3) is a mitochondrial deacetylase, which enhances respiratory chain function, reduces excessive ROS production, promotes PGC‐1α‐mediated mitochondrial biogenesis, and protects against oxidative stress via downstream targets like SOD2 [73]. Dual‐gene engineered exosomes co‐loading SIRT3 and insulin‐mimetic domains have demonstrated superior mitochondrial protection and metabolic improvement in myocardial ischemia–reperfusion models, so analogous strategies hold strong promise for reversing mitochondrial dysfunction [74].

For instance, miR‐29b‐3p can upregulate in aging‐associated IR and negatively regulate SIRT1 and insulin sensitivity [63]. It also can be knocked down using antagonists, siRNAs, or CRISPR interference at the MSC level, thereby reducing its loading into exosomes and blocking its inhibitory effects on SIRT1/insulin signalling [63].

Utilization of internal ribosome entry sites or 2A self‐cleaving peptides can construct bicistronic or multi‐cistronic vectors, which enable exosomes to load miR‐21, miR‐146a, and SIRT3 combined with anti‐inflammatory or metabolic modulators like Exendin‐4 or apelin [75]. Preconditioning of MSCs in hypoxia or cytokine exposure state further enhances cargo loading efficiency.

Synergizing genetic engineering with surface functionalization strategies significantly refines biodistribution, such as polyethylene glycol (PEG) or the conjugation of organ‐specific homing peptides, aptamers, and antibodies. Additionally, USP5@Exosome‐CP, a dual‐engineered vesicle co‐expressing CXCR4 and a P‐selectin‐binding peptide, has been developed to enhance tissue‐specific homing [76]. The functionalization of exosomes with DC‐specific aptamers has proven efficacy in a murine model of allergic airway inflammation, where sublingual delivery induced significant immunomodulatory effects and suppressed pathological symptoms [77]. An integrated nano‐therapeutic platform with a hepatocyte targeting antibody against ASGR1 can activate hepatocyte targeting and offer a promising translational approach for metabolic‐associated steatotic liver disease treatment [78].

These engineered strategies have consistently outperformed native MSC‐Exos in preclinical metabolic stress and diabetes complication models. Examples include superior glycemic control [36], enhanced insulin sensitivity [63], reversal of hepatic steatosis [37], and improved β‐cell preservation in HFD‐induced obese/IR mice [38]. Enriched miR‐21 or miR‐146a exosomes also exhibit stronger tissue repair and anti‐inflammatory effects in models of diabetic wound healing, neuropathy, and erectile dysfunction [45].

Overall, gene‐modified MSC‐Exos coordinate multi‐level interventions from signalling restoration to mitochondrial rescue to address the root causes of disease. This comprehensive approach is totally different from conventional single‐target drugs because the conventional methods are limited to symptom management [74]. While challenges regarding good manufacturing practice (GMP) production, stability, and regulatory standardization persist, gene‐engineering and delivery system design are paving the way for a paradigm shift. These advances make MSC‐Exos a transformative tool for disease modification rather than mere control [79].

## 5 Mitochondrial Crosstalk: MSC/MSC‐Exos, Mitochondria & IR

### 5.1 Mitochondrial Transfer From MSCs

MSCs can transfer functional mitochondria to stressed or injured cells, which relies on various ways, such as TNTs, gap junctions, or vesicles like exosomes [80]. Donating mitochondria to injured lung or endothelial cells rescues energy production and prevents tissue damage [51]. This mechanism is highly relevant to acute injury and chronic metabolic diseases; otherwise, mitochondrial failure is a common issue in insulin‐sensitive tissues [81].

For T2DM and IR, mitochondrial transfer from MSCs shows a promising strategy to correct bioenergetic deficits in key tissues like skeletal muscle, hepatocytes, and islets. According to this, mitochondrial donation can reshape metabolic reprogramming and alleviate oxidative stress in metabolic disease models [82].

### 5.2 Mitochondrial Health and Insulin Sensitivity

Mitochondria are essential for maintaining insulin sensitivity through governing substrate oxidation, ATP synthesis, and ROS regulation. In IR skeletal muscle, oxidative capacity declines, fatty acid oxidation varies, and the levels of ROS rise [83]. These defects are accompanied by disrupted mitochondrial dynamics (fusion/fission), impaired mitophagy, and a downregulated biogenesis pathway (PGC‐1α/NRF1/TFAM) [84]. Similarly, hepatic mitochondrial dysfunction drives excessive gluconeogenesis and steatosis, but in pancreatic β‐cells, it exacerbates stress‐induced apoptosis and hampers insulin secretion [85].

Glucose uptake and insulin signalling are influenced by the restoration of mitochondrial quality control, including biogenesis and ROS defence. MSCs can activate the SIRT1/FoxO3a pathway in pancreatic α‐cells within T2DM mice models, effectively mitigating mitochondrial dysfunction and normalizing glucagon levels [86]. These results reveal the importance of mitochondrial integrity for metabolic health and validate the therapeutic potential of targeting mitochondrial rescue in IR.

### 5.3 Engineered Exosomes for Mitochondrial Regulation

Engineered MSC‐Exos provide a sophisticated alternative to whole‐cell transplantation, and they are designed to support mitochondrial function. They deliver regulatory cargo like miRNAs and proteins that restore oxidative phosphorylation and mitigate oxidative stress [87]. Exosomes co‐loaded with SIRT3 and insulin can enhance respiration, glucose uptake, and oxygen consumption in ischemia–reperfusion injury [74].

While direct transfer of intact mitochondria via exosomes remains mechanistically debated (most evidence favours miRNA/protein mediated regulation rather than whole‐organelle delivery), MSC‐Exos enriched with antioxidative mediators or mitochondrial regulators have shown promise in protecting against metabolic and inflammatory stress in preclinical models of diabetes and related complications [88]. These approaches may indirectly support mitochondrial quality control by modulating PGC‐1α, AMPK, and SIRT pathways [89].

Although SIRT3 and other engineered mitochondrial regulators have shown promising results, most data originate from acute injury models (e.g., ischemia–reperfusion) [74], [90]. Their long‐term efficacy, safety, and actual mitochondrial rescue capacity in chronic IR settings have been insufficiently demonstrated.

### 5.4 Mitochondrial Crosstalk Mechanisms Relevant to IR

Mitochondrial dysfunction drives the transition from compensated IR to sustained hyperglycemia by impairing oxidative metabolism, increasing ROS, and disrupting signalling (e.g., excessive JNK activation, IRS serine phosphorylation) [91]. Key protective pathways include AMPK activation, PGC‐1α‐driven biogenesis, SIRT‐dependent deacetylation, and efficient mitophagy [92]. In multi‐organ IR, inter‐cellular mitochondrial crosstalk via TNTs, EVs, or paracrine factors may enable remote metabolic rescue [93] (Table 3).

**TABLE 3:** Key mitochondrial crosstalk mechanisms and their relevance to IR.

Mechanism	Description	Relevance to IR	References
Mitochondrial transfer (TNT/EV‐mediated)	MSCs transfer intact functional mitochondria to damaged cells via TNTs, vesicles, or direct cytoplasmic continuity	Mitigates mitochondrial oxidative stress and reduces metabolic stress in insulin‐target cells and tissues	[94]
Mitochondrial cargo in exosomes	Exosomes carry mitochondrial proteins, mt‐DNA, and mt‐RNA	Supports mitochondrial biogenesis, controls ROS, modulates mitophagy	[95, 96]
Gene‐engineered MSC‐Exos (e.g., SIRT3)	MSCs engineered to co‐load exosomes and insulin with mitochondrial regulators (SIRT3, antioxidant enzymes)	Improves mitochondrial respiration, reduces oxidative stress, enhances glucose uptake	[74]
Mitochondrial signalling modulation	MSC/MSC‐Exos activate SIRT1/FoxO3a, AMPK‐SIRT1‐PGC‐1α pathway	Enhances mitochondrial function and insulin sensitivity, reduces lipotoxicity and inflammation	[37, 86]

MSCs and exosomes help restore mitochondrial function by promoting biogenesis and mitophagy, and limiting ROS [97]. This strategy is further strengthened with pairing miRNA modulation, which offers a comprehensive framework to correct the signalling and organellar dysfunction characteristic of IR.

## 6 Gene‐Modified MSC‐Exos: Design, Strategies and Therapeutic Potential

### 6.1 MSC Modification Approaches

Genetic modification modifies MSCs and refines their exosome cargo for better targeting of IR and T2DM. Standard approaches use viral or non‐viral vectors to overexpress or silence specific genes, mainly focusing on miRNAs, mitochondrial regulators, and metabolic modulators.

Strategies address enriching exosomes with protective miRNAs and proteins. For example, overexpressing miR‐21 preserves β‐cells, while miR‐3075‐inspired constructs enhance insulin sensitivity [55, 64]. Additionally, incorporating proteins like SIRT3 targets insulin signalling and redox balance restoration [98]. Currently, a bicistronic construct or multi‐cistron makes it possible to package multiple therapeutic agents into a single exosome [74]. By combining elements like SIRT3 with targeting peptides or insulin‐mimetic domains, synergistic effects have been achieved in preclinical models of oxidative stress [74]. Priming MSCs through hypoxia, cytokine exposure, or small molecules significantly augments the bioactivity and loading capacity of their cargo. Modified MSCs to release Exendin‐4 or apelin strengthen their paracrine function in T2DM mouse models, which has successfully restored hepatic insulin sensitivity and mitochondrial function [99, 100].

### 6.2 Exosome Isolation, Characterization & Targeting Considerations

The ability to produce and characterize exosomes on a large scale can influence the clinical translation. The isolation technique revolves around ultracentrifugation, tangential flow filtration, or size‐exclusion chromatography [101]. After isolation, quality control is demonstrated by using nanoparticle tracking analysis, electron microscopy, and western blotting for CD9, CD63, and TSG101 [102]. Cargo stability, storage conditions, and in vivo biodistribution still remain critical challenges because miRNAs and proteins are susceptible to degradation in circulation.

Surface engineering modification with antibodies or targeting peptides improves organ‐specific delivery to main IR‐affected skeletal muscle, liver, and pancreatic islets [103]. Exosomes from adipose‐derived MSCs have been demonstrated to promote skeletal muscle regeneration [104]. Compared to unmodified MSC‐Exo, the aptamer and PEG modified exosomes have shown superior islet protection. This enhances a synergistic mechanism of stems including PEG prolonging systemic circulation; on the contrary, the aptamer ensures precise β‐cell homing [105] (Table 4).

**TABLE 4:** Comparison of methods for Gene‐Modified MSC‐derived exosomes.

Modification strategy	Methods	Main mechanism	Advantages	Disadvantages	References
Indirect modification (parental cell engineering)	Viral transduction (e.g., Lentivirus, Adenovirus)	Transducing MSCs with viral vectors carrying therapeutic genes; exosomes naturally package the expressed RNAs or proteins	(1) High transfection efficiency (2) Stable and long‐term expression (3) Suitable for complex genetic engineering	(1) Easy insertional mutagenesis (2) High cost and stringent biosafety level requirements (3) Potential for viral contamination in final product	[106]
	Non‐viral transfection (e.g., Plasmid transfection, Electroporation of cells)	Introducing plasmids into MSCs via liposomes or electroporation; exosomes inherit the modified cargo	(1) Safer profile compared to viral vectors (2) Lower cost and easier operation (3) Transient expression reduces long‐term risks	(1) Lower transfection efficiency than viral methods (2) Transient expression need repetition (3) Potential cytotoxicity to parental MSCs	[107]
Direct modification (post‐isolation engineering)	Electroporation	Applying electric pulses to exosomes to create temporary pores for gene entry (siRNA, miRNA, or plasmids)	(1) High loading efficiency for nucleic acids (2) Direct control over cargo dosage (3) Independent of parental cell status	(1) Disrupt exosome membrane integrity (2) Potential induction of exosome aggregation (3) Requires optimization to avoid cargo degradation	[108]
	Chemical transfection (e.g., Lipofectamine)	Using lipid‐based reagents to facilitate the entry of genetic material into isolated exosomes.	(1) Simple, rapid, and high‐throughput compatible (2) No need specialized electroporation equipment	(1) Difficult to separate exosomes from transfection reagent complexes (2) Reagents may keep contaminants	[109]
Surface display engineering (targeting modification)	Fusion protein expression (e.g., Lamp2b, CD63 fusions)	Genetically fusing targeting peptides/antibodies with exosome membrane proteins via parental cell transfection	(1) Achieves organ‐specific targeting (e.g., pancreas or muscle) (2) Stable present of targeting ligands	(1) Need precise genetic design (2) Fusion may affect protein folding or function	[110, 111]

### 6.3 Potential Therapeutic Design for IR

Constructing an optimized gene‐modified MSC‐Exo platform for IR or T2DM requires a multifaceted approach. Ideally, exosomes derived from hUC‐MSCs or BMMSCs act as the foundation, which offers high yield and minimal immunogenicity. Genetic engineering strategies can then be applied to upregulate protective agents like miR‐21 and SIRT3 while suppressing miR‐29b‐3p [63, 112, 113]. To maximize efficacy, the platform should incorporate multi‐functional payloads and tissue‐specific surface ligands in order to ensure targeted delivery to metabolic organs following validated dosing regimens.

In preclinical studies, engineered platforms with apelin‐loaded or SIRT3‐overexpressing exosomes have more superior efficacy than native MSC‐Exos [114]. The modified function of vesicles by reactivating the SIRT1 signalling pathway directly increases insulin sensitivity [115]. Therefore, the therapeutic benefits include improving mitochondrial respiration and glucose homeostasis, and attenuating oxidative stress and systemic inflammation.

Nevertheless, current research of enhanced efficacy focuses on small‐scale rodent models. The rigorous validation of engineered constructs has never undergone in large animal models. Furthermore, the clinical application is obstructed due to manufacturing reproducibility and the systematic assessment of long‐term safety and immunogenicity.

## 7 Challenges, Considerations and Future Directions

### 7.1 Manufacturing Standardization and Scalability

The clinical translation of MSC‐Exo therapy has been hindered by manufacturing. Current isolation protocols from ultracentrifugation to size‐exclusion chromatography compromise production and reproducibility, which are still a challenge. These standardization challenges are further exacerbated by the genetic modification process itself; meanwhile, variability in transfection efficiency and cargo loading brings an additional layer of complexity [116, 117]. So, it is difficult to achieve the consistent production of standard exosome formulations.

Improvement in bioreactor systems has boosted production, but quality assurance is inadequate for IR applications. There is a lack of validated efficacy assessment for IR relevant functions like insulin sensitivity and anti‐inflammatory responses. Thus, MISEV2023 standards are insufficient, so it is essential for validating product criteria for clinical‐grade application.

### 7.2 Safety, Immunogenicity, and Long‐Term Effects

The genetic engineering of MSC‐Exos induces novel risks accompanied with warrant caution. The use of viral vectors raises insertional mutagenesis and unintended immune activation; in the meantime, it is ambiguous about long‐term oncogenic potential [118]. Furthermore, therapeutic efficacy is constrained by altered pharmacokinetics. The biodistribution analyses reveal a strong tendency for systemic clearance through the liver and spleen, but the bioavailability and retention of exosomes in skeletal muscle and pancreatic islets are limited.

A critical barrier to clinical translation is the lack of data on long‐term safety. Specifically, the risks are associated with repeated dosing, chronic immunogenicity, and the potential off‐target effects of overexpressed agents like SIRT3 [119, 120]. Therefore, it is necessary to establish toxicology assessments in large animal models in order to support prolonged human clinical trials.

### 7.3 Biodistribution, Targeting, and Multi‐Organ Delivery

The systemic nature of IR impairs the function of skeletal muscle, adipose tissue, the liver, and pancreatic islets. Therefore, exosome delivery remains a formidable technical challenge. The surface engineering strategies have demonstrated preliminary success in enhancing targeting specificity, such as PEGylation, the use of homing peptides and aptamers, but their validation leaves a significant gap in translational evidence [121].

A primary pharmacokinetic hurdle is the rapid sequestration of exosomes by the mononuclear phagocyte system. This process limits their bioavailability within peripheral insulin‐sensitive tissues. Whether engineered targeting can achieve sufficiently high and durable accumulation in multiple IR‐affected organs simultaneously still needs to be convincingly demonstrated.

### 7.4 Mechanistic Elucidation and Biomarker Development

Many reported therapeutic effects are based on correlative rather than causative evidence. The relative contribution of individual miRNAs versus proteins, direct versus indirect mitochondrial effects, and short‐range versus systemic signalling in IR remains incompletely apparent. Otherwise, reliable pharmacodynamic biomarkers that can predict responders and monitor therapeutic efficacy in real time are incompletely characterized.

Multi‐omics approaches like single‐cell RNA‐seq, metabolomics, and mitochondrial functional profiling combined with advanced imaging should be prioritized to elucidate cargo‐mediated mechanisms and identify robust translational biomarkers [122].

### 7.5 Regulatory and Clinical Translation Considerations

Gene‐modified MSC‐Exos are classified as advanced therapy medicinal products and subjected to stringent regulatory requirements. As of 2025, exosome‐based therapies have encompassed early‐phase clinical trials mainly for wound healing, graft‐versus‐host disease, and certain complications of diabetes, but no large‐scale trials have specifically targeted systemic IR [123].

The field currently suffers from a lack of comparative data relative to standard‐of‐care drugs, as well as uncertain dosing protocols and long‐term prognostic benefits. Future research must move beyond preliminary validation by implementing adaptive trial designs. A strategy would start from defined patient subsets, particularly in those patients with prediabetes or early T2DM with mitochondrial dysfunction.

### 7.6 Future Directions

The next‐generation engineering strategies should construct exosomes that integrate mitochondrial regulators like SIRT3 with precise tissue‐targeting ligands. In a mechanistic level, the application of advanced tools like spatial transcriptomics and organoid models are important to clarify how exosome cargos regulate inter‐organ crosstalk. When exosomes act as drug carriers, they can load paclitaxel to inhibit multiple drug resistance tumours and further enhance the potency of drugs [124]. Therefore, exploring the synergistic ability of these exosomes with established pharmacotherapies could unlock superior clinical outcomes for IR therapy.

Gene‐modified MSC‐Exos offer a multi‐target approach to IR. Their clinical application is currently obstructed by scalable production, targeted delivery, and mechanistic depth [125]. Overcoming these barriers needs rigorous, well‐controlled translational investigation, so how to develop this technology from experiment to a genuine disease‐modifying therapy is still a challenge.

## 8 Conclusion

IR is the pathogenic base of T2DM, metabolic liver disease, obesity, and broader cardiometabolic disorders. Although conventional pharmacotherapies effectively manage glycemic indices, they offer modest improvements in systemic insulin sensitivity. These standard interventions are incapable of correcting the complexity of the impaired signalling pathways, chronic inflammatory states, ectopic lipid burden, and mitochondrial dysregulation.

MSC‐Exos represent a cell‐free alternative, which has the paracrine benefits of their parent cells without the logistical complexities of cell therapy. In the preclinical phase, it has been proved that these vesicles can restore insulin signalling, mitigate inflammatory pathways, preserve β‐cell integrity, and revitalize mitochondrial function through the horizontal transfer of bioactive cargo. To further amplify these effects, genetic engineering strategies including the enrichment of miR‐21 or miR‐146a and the overexpression of SIRT3 have been successfully utilized in various models of metabolic syndrome.

**FIGURE 1:**
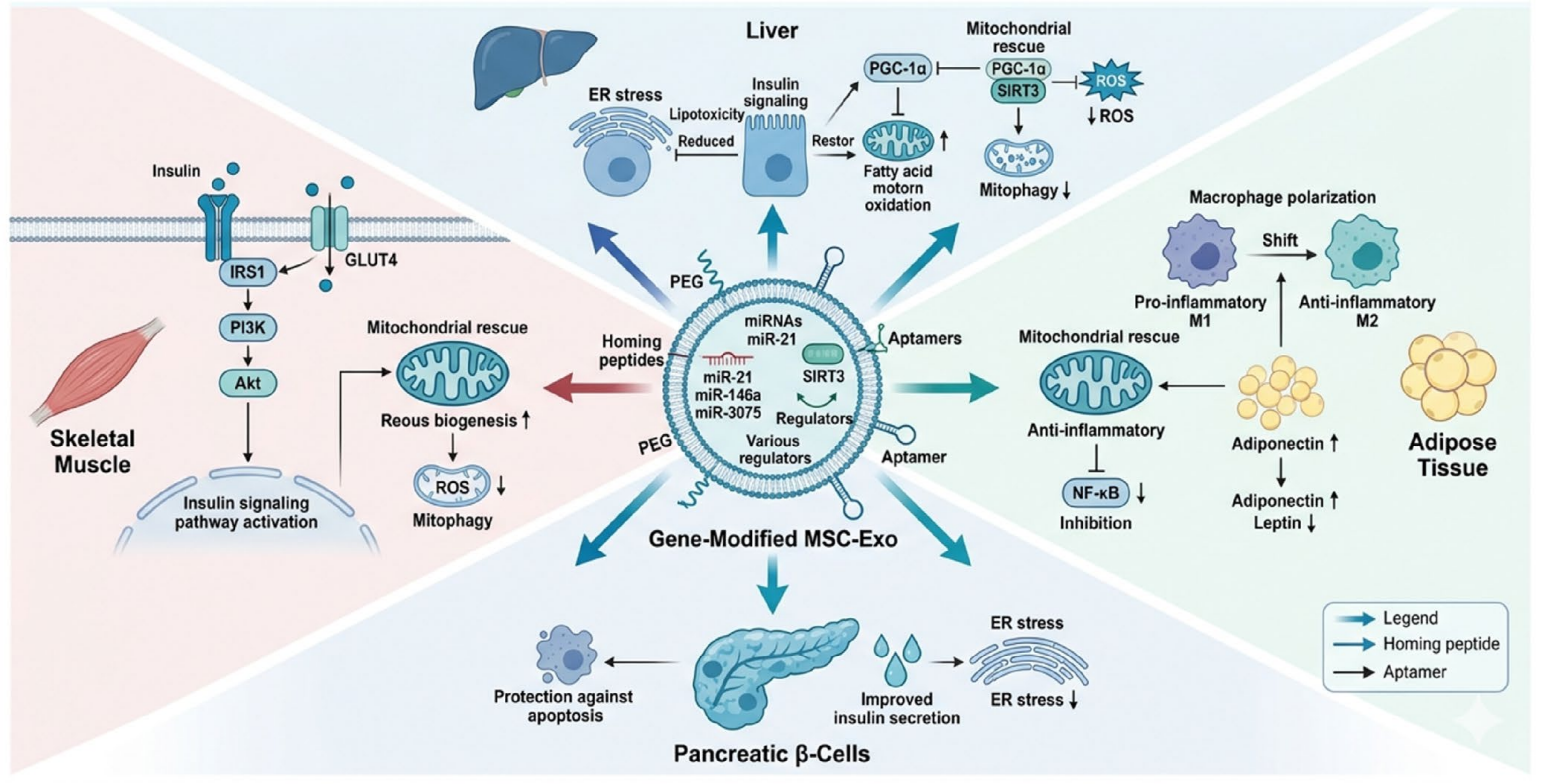
Schematic illustration of the multi‐target therapeutic mechanisms of gene‐modified MSC‐Exos in IR.

Gene‐modified MSC‐Exos constitute a complicated therapeutic framework. This technique is relevant to a holistic mechanism that targets the core pathologies of IR, including revitalizing insulin signalling, β‐cell integrity, and mitochondrial function, inhibiting inflammatory pathways. However, this research is still at an early preclinical stage. Substantial gaps in manufacturing standardization, in vivo targeting efficiency, and clinical‐grade evidence must be resolved through rigorous large‐animal studies and biomarker‐driven investigations. By improving the engineering through utilizing the unique characteristics of exosomes, gene‐modified MSC‐Exos can be transferred from experimental tools to genuine disease‐modifying therapies for IR and its metabolic sequelae (Figure 1).

## Author Contributions

**Yuanyuan Zhang:** conceptualization, validation. **Baodong Gao:** writing – review and editing, supervision. **Yuan Xing:** data curation, investigation, resources. **Xiangying Li:** writing – review and editing, visualization. **Xiaoqin Ha:** project administration, writing – review and editing. **Bing Li:** software, writing – review and editing, methodology. **Qinghua Jia:** writing – original draft, funding acquisition, formal analysis.

## Funding

This research was funded by Lanzhou Science and Technology Plan Project (2023‐ZD‐166) and Gansu Province Health Industry Research Plan Project (GSWSKY2023‐06).

## Conflicts of Interest

The authors declare no conflicts of interest.

## Data Availability

The data that support the findings of this study are available from the corresponding author upon reasonable request.
